# Microbiome Management for the 21st Century and Beyond

**DOI:** 10.1128/msystems.00760-21

**Published:** 2021-08-31

**Authors:** Andrew J. Hryckowian

**Affiliations:** a Department of Medicine, University of Wisconsin—Madison, Madison, Wisconsin, USA; b Department of Medical Microbiology and Immunology, University of Wisconsin—Madison, Madison, Wisconsin, USA

**Keywords:** antibiotic resistance, bacterial pathogens, bacteriophages, dietary fiber, microbiome

## Abstract

As we learn about the sophisticated and far-reaching impacts that our resident microbiomes have on our biology, it is apparent that the tools we have for managing our microbiomes are rudimentary at best. For example, though antibiotics rid our microbiomes of bacterial pathogens, they target pathogens and commensals alike. Additional approaches, such as fecal microbiome transplant, seem to restore a healthy microbiome in some applications, but the mechanisms underlying this treatment and its long-term effects are poorly understood. Here, I discuss my laboratory’s research, which uses two major drivers of gut microbiome ecology, diet and bacteriophages, as tools to develop new concepts and approaches for managing microbiomes. I speculate on the anticipated impacts of this research and how it will influence the way that we treat the kaleidoscope of microbe-microbe and microbe-host interactions central to our health.

## COMMENTARY

Multicellular life evolved in, and continues to inhabit, a world dominated by microorganisms. A poignant illustration of humanity’s place in this microbial world is our relationship with the microbial communities (microbiomes) that inhabit our bodies. It seems that no part of our biology is untouched by the microbial majority. For example, our microbiomes influence our susceptibility to infectious diseases (1), immune development (2), and energy harvest (3). These observations highlight the intensifying and largely unmet need to understand and manage our microbiomes to better control their impacts on our health.

As we gain a greater appreciation for the ways our microbiomes impact our biology, it is increasingly clear that we are deficient in precision tools for managing these microbial communities. For example, antibiotics target pathogens and beneficial bacteria alike. As such, antibiotics alter our microbiomes (sometimes permanently), and antibiotic-mediated microbiome disruption is now recognized as a key player in the rising incidence of immune dysregulation and chronic inflammatory conditions, often termed “Western diseases” (e.g., asthma, atopic dermatitis) (2, 4, 5). In addition, widespread use of antibiotics has set the stage for the antibiotic resistance crisis, where many deadly pathogens are becoming increasingly resistant to our arsenal. This crisis is further exacerbated by several major pharmaceutical companies recently deciding to abandon their antibiotic discovery and development pipelines (6). Alternative strategies to antibiotics, such as fecal microbiome transplant (FMT), have been implemented and show promise, especially in treating recurrent infection by the diarrheal pathogen Clostridioides difficile (7). However, a mechanistic understanding of why FMT works remains elusive, although simplified bacteriotherapies based on FMT are on the horizon (8), suggesting that a mechanistic dissection of the FMT concept (and, by extension, a reduction of off-target effects and robust application to other diseases) may be within reach.

All of this raises important questions. Can we use the confluence of the antibiotic resistance crisis and our expanding understanding of microbiomes as an opportunity to devise new strategies for managing microbiomes? What are the most effective ways of developing these strategies? To begin to address these questions in my laboratory, we use controlled experimental systems that leverage two major drivers of gut microbiome community dynamics: dietary fiber and bacteriophages.

## WHAT CAN WE LEARN FROM MAJOR DRIVERS OF GUT MICROBIOME STRUCTURE AND FUNCTION?

### Dietary fiber.

Our health relies on our gut microbiomes’ ability to metabolize dietary fiber. The human genome encodes 17 carbohydrate-degrading enzymes, leaving us unable to extract calories from many plant-derived polysaccharides (dietary fiber), which subsequently transit to the distal gut and serve as the preferred food source for many members of our gut microbiome. Indeed, the distal gut microbiomes of humans and other animals encode tens of thousands of carbohydrate-degrading enzymes capable of breaking various polysaccharide linkages (9). This metabolism of dietary fiber supports a diverse community of microorganisms in the distal gut. Furthermore, fiber metabolism has a number of beneficial effects on the host, ranging from maintenance of gut anaerobiosis to regulation of the immune system (10). When the microbiome is deprived of fiber, this microbial community increases its consumption of intestinal mucus (11). This metabolic switch is accompanied by significant changes in the gut microbiome community structure and elevated inflammation in mice (12, 13). So, although the gut microbiome provides many benefits to the host, the dysbiosis associated with a fiber-deficient diet is a driver of inflammation in the gut in experimental models and in humans.

**FIG 1:**
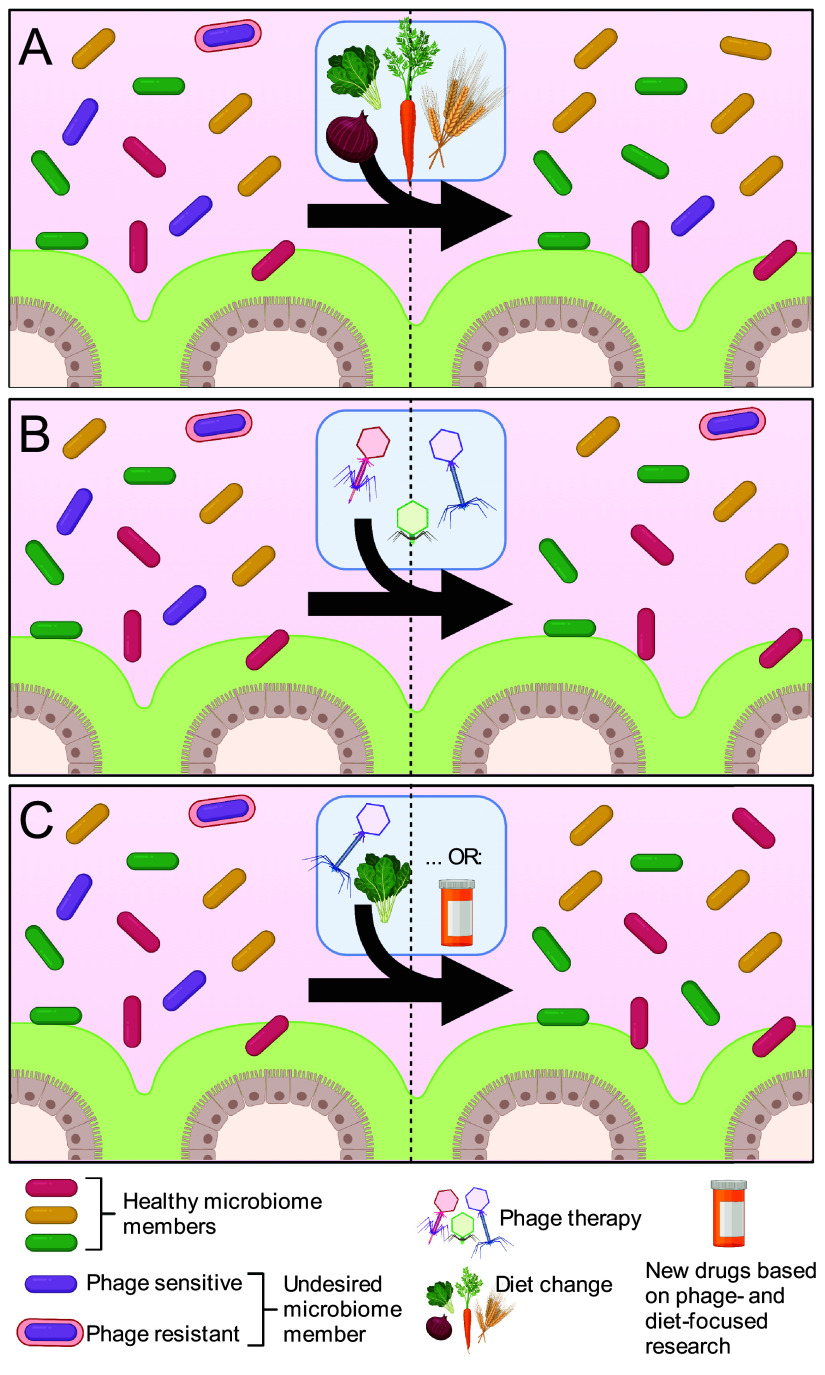
A general overview of diet- and phage-based microbiome management strategies. (A) Dietary intervention impacts the microbiome by altering the metabolic landscape of the gut and can be used to simultaneously increase the abundance of healthy microbiome members and decrease the abundance of undesired microbiome members. However, key questions remain relating to which specific dietary components (e.g., which fiber types) impact these phenotypes and the degree to which they are generalizable across individuals and across disease states. (B) Phages exhibit a high degree of specificity toward target bacteria and can be used to reduce the abundance of undesired microbiome members. However, the ways in which host- and microbiome-determined variables influence the efficacy of phage therapy and ways to overcome phage resistance *in vivo* remain poorly defined. (C) It is possible that by combining diet- and phage-based approaches, a greater degree of precision and accuracy can be attained. For example, by using diet to force phenotypic changes in target bacteria, phage therapy may be more effective. By extension, I hypothesize that by learning the molecular mechanisms and genetic circuitry underlying how major drivers of microbiome structure and function (e.g., phages and diet) impact microbial communities, new drugs or other precision approaches can be developed to target specific microbe-microbe or microbe-host interactions. Figure created with BioRender (BioRender, Toronto, ON, Canada).

A growing body of literature supports that in addition to promoting immune homeostasis, fiber mitigates infection by gastrointestinal pathogens (Fig. 1A). For example, two independent human trials showed that fiber aids in host recovery from infection by the enteric pathogen *Shigella* (14, 15). Additional work in animal models of infection demonstrated that fiber metabolism by the microbiome is an important deterrent to bacterial pathogens like *Citrobacter*, *Salmonella*, and C. difficile (16–18). Therefore, a deeper understanding of the impacts of dietary fiber on the ecology of the gut microbiome will enable the development of diet-based strategies to mitigate a variety of diseases.

Although dietary fiber is one of the most tractable ways to alter the structure and function of the gut microbiome, gaps in knowledge of the molecular mechanisms underlying these changes hinder translation. My previous work showed that dietary fiber influences infection with the diarrheal pathogen C. difficile in a murine model: mice fed high-fiber diets clear C. difficile infection (CDI) within days while mice fed low-fiber diets have persistent CDI. In particular, short-chain fatty acids (SCFAs) (the major metabolic end products of dietary fiber metabolism by the gut microbiome) impact C. difficile fitness and virulence (18). In my laboratory, we are working to uncover the molecular mechanisms and genetic circuitry underlying how C. difficile, other pathogens, and members of the microbiome respond to dietary fiber and SCFAs.

### Bacteriophages.

Bacteriophages (phages) are viruses that infect bacteria. Phages are the most abundant biological entities on the planet (an estimated 10^31^ phage particles are present on Earth) and in the gastrointestinal tracts of humans (up to 10^12^ particles per gram of human stool) (19, 20). Phages provide key evolutionary selective pressures in ecosystems across the globe. Though mounting evidence suggests the importance of phages in human microbiomes (21), methods of data generation and analysis routinely used in microbiome science (e.g., metagenomics) neglect important aspects of phage biology. For example, the overwhelming majority of phages detected in human gut metagenomes cannot be classified taxonomically, linked to any bacterial hosts, or be assigned an ecological role in the gut. Furthermore, only a few tractable experimental systems for studying gut-resident phages exist, leaving many basic questions unanswered.

Regardless, many scientists and clinicians believe that “phage therapy” will be an important tool in our recovery from the growing threats imposed by antibiotic-resistant pathogens. Importantly, unlike antibiotics, phages target bacteria with strain-level specificity and can be used without inflicting collateral damage on beneficial microbes (5). Indeed, phage therapy is routine in some parts of the world, and anecdotal evidence from places like the Eliava Institute in Tbilisi, Georgia, provides hope that phage therapy is a viable treatment option for bacterial infections. This excitement is reinforced by two recent high-profile compassionate use cases in the United States and England, where drug-resistant *Acinetobacter* and *Mycobacterium* infections were resolved via phage therapy (22, 23).

Despite past triumphs and promise for the future, phage therapy is inconsistently effective. This is in large part due to an incomplete understanding of how phages behave in complex, host-associated microbial communities and is perpetuated by a deficiency of tractable experimental systems to study phages in host-associated microbial ecosystems. Previously, my work with the prominent human gut symbiont Bacteroides thetaiotaomicron integrated experimental and computational approaches to reveal determinants of phage-bacterial interactions in the gut (24, 25). In my laboratory, we are building on this previous work with B. thetaiotaomicron*-*infecting phages and are expanding into work with phages infecting other diverse gut microbes. Together, these efforts will provide much-needed experimental systems that will be used to understand the phage-, bacterium-, and host-determined variables that dictate phage infection and resistance in the gut (Fig. 1B).

### Anticipated synergy between diet- and phage-focused work.

As our work progresses, I expect that deeper connections between phage-based and diet-based projects will emerge. For example, based on my previous work and work from others, we know that capsular polysaccharides (CPS) are a major determinant of phage tropism (24, 25). The expressions of CPS are driven by environmental cues, such as host diet (11). Therefore, I hypothesize that diet-driven changes in expression of CPS and other surface-exposed epitopes may dictate whether a particular phage is able to infect its host in the gut. Future investigation into these and other diet-by-phage effects (e.g., nutrients that impact the lytic-lysogenic switch or influence localization of bacteria) will hone strategies for phage therapy, dietary recommendations, or allow for the development of combination therapies (Fig. 1C).

## NEXT STEPS AND IMPACTS

With our foci on dietary fiber and phages, we will learn foundational rules that govern host-microbiome relationships. The most logical outcome of our work will be a refined view of important facets of microbiome dynamics (e.g., microbiome resilience, stability, invasibility). We will apply these concepts to build precise and reproducible approaches to manage microbiomes (Fig. 1) and positively impact gastrointestinal diseases. Our diet-focused work will add to the growing examples of how our food influences our health. Beyond the obvious benefit of bolstering the advice for humans to eat more fiber, we will hone humans’ understanding of which (and under what circumstances) specific fiber types are important. For example, do all fiber types clear C. difficile infection? Are these fiber types generalizable to all individuals? Which types of patient data will physicians use to make decisions about which fiber types to recommend? Can these findings be generalized to other pathogens? Similarly, our phage-focused work will help to bring phage therapy out of obscurity and into the age of precision medicine. By isolating phages that infect phylogenetically diverse gut microbes, we will build model systems to understand the roles and identities of phages in the gut and build the understanding necessary for engineering phages to target specific bacteria, to localize phages to desired niches in the gut, or to impact the host immune system in prescribed ways. In addition to targeting pathogens, I envision that phages will be used to remodel disease-associated microbiomes (e.g., those associated with inflammatory bowel disease), either by directly removing keystone species within these communities or by influencing specific facets of the host immune response. By extension, I anticipate that as we continue our work, we will identify key players (e.g., microbiome members, host immune pathways) involved in the transitions between healthy and diseased states that will serve as targets for new generations of microbiome-managing drugs (Fig. 1C). Importantly, collaborations with basic, translational, and clinical research colleagues will help to build the knowledge and wisdom necessary to develop and apply alternative therapeutics (e.g., phages, dietary intervention, or new drugs) while being better stewards of existing ones (e.g., antibiotics).

## References

[1] Hryckowian AJ, Pruss KM, Sonnenburg JL. 2017. The emerging metabolic view of Clostridium difficile pathogenesis. Curr Opin Microbiol 35:42–47. doi:10.1016/j.mib.2016.11.006. 27997854 PMC5474191

[2] Belkaid Y, Hand TW. 2014. Role of the microbiota in immunity and inflammation. Cell 157:121–141. doi:10.1016/j.cell.2014.03.011. 24679531 PMC4056765

[3] Fischbach MA, Sonnenburg JL. 2011. Eating for two: how metabolism establishes interspecies interactions in the gut. Cell Host Microbe 10:336–347. doi:10.1016/j.chom.2011.10.002. 22018234 PMC3225337

[4] Aversa Z, Atkinson EJ, Schafer MJ, Theiler RN, Rocca WA, Blaser MJ, LeBrasseur NK. 2021. Association of infant antibiotic exposure with childhood health outcomes. Mayo Clinic Proc 96:66–77. doi:10.1016/j.mayocp.2020.07.019. PMC7796951 33208243

[5] Dethlefsen L, Huse S, Sogin ML, Relman DA. 2008. The pervasive effects of an antibiotic on the human gut microbiota, as revealed by deep 16S rRNA sequencing. PLoS Biol 6:e280. doi:10.1371/journal.pbio.0060280. 19018661 PMC2586385

[6] Hu C. 21 July 2018. Pharmaceutical companies are backing away from a growing threat that could kill 10 million people a year by 2050. Business Insider. https://www.businessinsider.com/major-pharmaceutical-companies-dropping-antibiotic-projects-superbugs-2018-7.

[7] van Nood E, Vrieze A, Nieuwdorp M, Fuentes S, Zoetendal EG, de Vos WM, Visser CE, Kuijper EJ, Bartelsman JFWM, Tijssen JGP, Speelman P, Dijkgraaf MGW, Keller JJ. 2013. Duodenal infusion of donor feces for recurrent Clostridium difficile. N Engl J Med 368:407–415. doi:10.1056/NEJMoa1205037. 23323867

[8] McGovern BH, Ford CB, Henn MR, Pardi DS, Khanna S, Hohmann EL, O’Brien EJ, Desjardins CA, Bernardo P, Wortman JR, Lombardo M-J, Litcofsky KD, Winkler JA, McChalicher CWJ, Li SS, Tomlinson AD, Nandakumar M, Cook DN, Pomerantz RJ, Auninš JG, Trucksis M. 2021. SER-109, an investigational microbiome drug to reduce recurrence after Clostridioides difficile infection: lessons learned from a phase 2 trial. Clin Infect Dis 72:2132–2140. doi:10.1093/cid/ciaa387. 32255488 PMC8204772

[9] Sonnenburg ED, Sonnenburg JL. 2014. Starving our microbial self: the deleterious consequences of a diet deficient in microbiota-accessible carbohydrates. Cell Metab 20:779–786. doi:10.1016/j.cmet.2014.07.003. 25156449 PMC4896489

[10] Byndloss MX, Olsan EE, Rivera-Chávez F, Tiffany CR, Cevallos SA, Lokken KL, Torres TP, Byndloss AJ, Faber F, Gao Y, Litvak Y, Lopez CA, Xu G, Napoli E, Giulivi C, Tsolis RM, Revzin A, Lebrilla CB, Bäumler AJ. 2017. Microbiota-activated PPAR-gamma signaling inhibits dysbiotic Enterobacteriaceae expansion. Science 357:570–575. doi:10.1126/science.aam9949. 28798125 PMC5642957

[11] Sonnenburg JL, Xu J, Leip DD, Chen C-H, Westover BP, Weatherford J, Buhler JD, Gordon JI. 2005. Glycan foraging in vivo by an intestine-adapted bacterial symbiont. Science 307:1955–1959. doi:10.1126/science.1109051. 15790854

[12] Kashyap PC, Marcobal A, Ursell LK, Larauche M, Duboc H, Earle KA, Sonnenburg ED, Ferreyra JA, Higginbottom SK, Million M, Tache Y, Pasricha PJ, Knight R, Farrugia G, Sonnenburg JL. 2013. Complex interactions among diet, gastrointestinal transit, and gut microbiota in humanized mice. Gastroenterology 144:967–977. doi:10.1053/j.gastro.2013.01.047. 23380084 PMC3890323

[13] Earle KA, Billings G, Sigal M, Lichtman JS, Hansson GC, Elias JE, Amieva MR, Huang KC, Sonnenburg JL. 2015. Quantitative imaging of gut microbiota spatial organization. Cell Host Microbe 18:478–488. doi:10.1016/j.chom.2015.09.002. 26439864 PMC4628835

[14] Rabbani GH, Ahmed S, Hossain MI, Islam R, Marni F, Akhtar M, Majid N. 2009. Green banana reduces clinical severity of childhood shigellosis: a double-blind, randomized, controlled clinical trial. Pediatr Infect Dis J 28:420–425. doi:10.1097/INF.0b013e31819510b5. 19319017

[15] Álvarez-Acosta T, León C, Acosta-González S, Parra-Soto H, Cluet-Rodriguez I, Rossell MR, Colina-Chourio JA. 2009. Beneficial role of green plantain [Musa paradisiaca] in the management of persistent diarrhea: a prospective randomized trial. J Am College Nutr 28:169–176. doi:10.1080/07315724.2009.10719768. 19828902

[16] Desai MS, Seekatz AM, Koropatkin NM, Kamada N, Hickey CA, Wolter M, Pudlo NA, Kitamoto S, Terrapon N, Muller A, Young VB, Henrissat B, Wilmes P, Stappenbeck TS, Núñez G, Martens EC. 2016. A dietary fiber-deprived gut microbiota degrades the colonic mucus barrier and enhances pathogen susceptibility. Cell 167:1339–1353. doi:10.1016/j.cell.2016.10.043. 27863247 PMC5131798

[17] Jacobson A, Lam L, Rajendram M, Tamburini F, Honeycutt J, Pham T, Van Treuren W, Pruss K, Stabler SR, Lugo K, Bouley DM, Vilches-Moure JG, Smith M, Sonnenburg JL, Bhatt AS, Huang KC, Monack D. 2018. A gut commensal-produced metabolite mediates colonization resistance to Salmonella infection. Cell Host Microbe 24:296–307. doi:10.1016/j.chom.2018.07.002. 30057174 PMC6223613

[18] Hryckowian AJ, Van Treuren W, Smits SA, Davis NM, Gardner JO, Bouley DM, Sonnenburg JL. 2018. Microbiota-accessible carbohydrates suppress Clostridium difficile infection in a murine model. Nat Microbiol 3:662–669. doi:10.1038/s41564-018-0150-6. 29686297 PMC6126909

[19] Brussow H, Hendrix RW. 2002. Phage genomics: small is beautiful. Cell 108:13–16. doi:10.1016/S0092-8674(01)00637-7. 11792317

[20] Hoyles L, McCartney AL, Neve H, Gibson GR, Sanderson JD, Heller KJ, van Sinderen D. 2014. Characterization of virus-like particles associated with the human faecal and caecal microbiota. Res Microbiol 165:803–812. doi:10.1016/j.resmic.2014.10.006. 25463385

[21] Duerkop BA, Kleiner M, Paez-Espino D, Zhu W, Bushnell B, Hassell B, Winter SE, Kyrpides NC, Hooper LV. 2018. Murine colitis reveals a disease-associated bacteriophage community. Nat Microbiol 3:1023–1031. doi:10.1038/s41564-018-0210-y. 30038310 PMC6112176

[22] Dedrick RM, Guerrero-Bustamante CA, Garlena RA, Russell DA, Ford K, Harris K, Gilmour KC, Soothill J, Jacobs-Sera D, Schooley RT, Hatfull GF, Spencer H. 2019. Engineered bacteriophages for treatment of a patient with a disseminated drug-resistant Mycobacterium abscessus. Nat Med 25:730–733. doi:10.1038/s41591-019-0437-z. 31068712 PMC6557439

[23] Schooley RT, Biswas B, Gill JJ, Hernandez-Morales A, Lancaster J, Lessor L, Barr JJ, Reed SL, Rohwer F, Benler S, Segall AM, Taplitz R, Smith DM, Kerr K, Kumaraswamy M, Nizet V, Lin L, McCauley MD, Strathdee SA, Benson CA, Pope RK, Leroux BM, Picel AC, Mateczun AJ, Cilwa KE, Regeimbal JM, Estrella LA, Wolfe DM, Henry MS, Quinones J, Salka S, Bishop-Lilly KA, Young R, Hamilton T. 2017. Development and use of personalized bacteriophage-based therapeutic cocktails to treat a patient with a disseminated resistant Acinetobacter baumannii infection. Antimicrob Agents Chemother 61:e00954-17. doi:10.1128/AAC.00954-17. 28807909 PMC5610518

[24] Porter NT, Hryckowian AJ, Merrill BD, Fuentes JJ, Gardner JO, Glowacki RWP, Singh S, Crawford RD, Snitkin ES, Sonnenburg JL, Martens EC. 2020. Phase-variable capsular polysaccharides and lipoproteins modify bacteriophage susceptibility in Bacteroides thetaiotaomicron. Nat Microbiol 5:1170–1181. doi:10.1038/s41564-020-0746-5. 32601452 PMC7482406

[25] Hryckowian AJ, Merrill BD, Porter NT, Van Treuren W, Nelson EJ, Garlena RA, Russell DA, Martens EC, Sonnenburg JL. 2020. Bacteroides thetaiotaomicron-infecting bacteriophage isolates inform sequence-based host range predictions. Cell Host Microbe 28:371–379. doi:10.1016/j.chom.2020.06.011. 32652063 PMC8045012

